# Four Extremity Amputation and Bionic Prosthesis Supply after Disseminated Intravascular Coagulation: A Follow-Up on Functionality and Quality of Life after Bionic Prosthesis Supply

**DOI:** 10.29252/wjps.8.2.146

**Published:** 2019-05

**Authors:** Dennis Werner, Seyed Arash Alawi

**Affiliations:** Department of Plastic, Aesthetic, Hand and Reconstructive Surgery, Hannover Medical School, Hannover, Germany

**Keywords:** Disseminated intravascular coagulopathy, Amputation, Reconstruction, Bionic, Myoelectric, Prosthesis, Rehabilitation

## Abstract

**BACKGROUND:**

Disseminated intravascular coagulopathy (DIC) is a rare symptom complex that causes embolisms within the microvasculature and extensive necrosis of the skin and the acres. During surgical decision-making, preserving functionally important structures must be weighed against radical debridement. The aim was to analyze functional recovery and quality of life of patients sustaining amputations from disseminated intravascular coagulopathy and supplied with bionic prostheses.

**METHODS:**

A monocentric, retrospective review of patients with disseminated intravascular coagulopathy after sepsis was conducted from 2016 to 2018. After initial reconstruction and intensive care treatment, patients were provided with bionic prosthetic devices. A follow-up survey measuring function and quality of life was performed.

**RESULTS:**

Three patients (mean: 45 years; median: 50 years) were analyzed. The first necrectomy and amputation were performed, on average, after >4 weeks post-symptom onset. All patients required re-amputation, averaging two or one re-amputations in the right or left upper extremity, respectively, and one in ​​the lower extremities. On average, 12 operations for reconstruction of skin defects were required (x͂=8). On average, patients tolerated their prostheses for 5.67 h per day. Satisfaction metrics were either sufficient (SF-36, x̅=69) or moderate (TAPES-R, x̅=4.7). Physical skills were rated poor to fair (average TAPES-R=2.67).

**CONCLUSION:**

Supplying bionic prostheses after DIC yielded sufficient to moderate results. However, prothesis weight, signal transmission disorders, and repeated functional failures were suboptimal. For extensive stump scarring, implantable signal electrodes may improve signal transmission.

## INTRODUCTION

Disseminated intravascular coagulopathy (DIC) is a rare symptom complex that causes embolisms within the microvasculature and extensive necrosis of the skin and the acres.^1^ During surgical decision-making, preserving functionally important structures must be weighed against radical debridement. These special symptom complexes require an interdisciplinary approach and individually tailored therapy in specialized centers to avoid high mortality, which is stated to be between 22% to 46%. However, the mortality of patients with DIC decreased from 65% in 1992 to 46% in 2012.^2^^,^^3^

DIC is characterized by the pathologically excessive formation of thrombin and fibrin in the systemic circulation in the context of sepsis, shock, severe tissue damage, or malignant tumors,^4^ leading to increased platelet aggregation and the consumption of coagulation factors.^5^ Rapid DIC, developing over hours to days, often causes bleeding, whereas the slow form, developing over weeks or months, results in predominantly thrombotic and embolic events. Diagnostically, DIC is characterized by thrombocytopenia, prolonged partial thromboplastin time (PTT), increased D-dimers and decreased plasma fibrinogen levels.^6^

Therefore, it is crucial to administer platelet concentrates and fresh frozen plasma to control bleeding. For the slow form of DIC using heparin can prevent venous thrombi. Full heparinization with a target aPTT of 45-60 is associated with reduced mortality in patients with sepsis.^1^^,^^6^ An overview of DIC characteristics is shown in Table 1. Further therapy consists of removing underperfused and avital tissue after demarcation. The necrectomy of the avital tissue and the amputation of acral necrosis after demarcation is necessary in DIC as well as in PF.^7^

**Table 1:** Overview of the clinical parameters of disseminated intravascular coagulopathy

**Variable**	**Disseminated intravascular coagulation**
Pathogenesis	- Systemic shock, severe tissue damage or malignant tumors - Excessive formation of thrombin and fibrin in the systemic circulation - Emboli within the microvasculatureIncreased platelet aggregation and consumption of coagulation factors
Findings	- From bleeding to thrombotic and embolic events - Violet-bluish, partly hemorrhagic skin lesions - Full-thickness necrosis of the skin and acres
Diagnostics	- Blood collection with thrombocytopenia, prolonged PTT, increased - D-Dimeric and reduced fibrinogen plasma levels
Therapy	- Administration of platelet concentrate and coagulation factors due to frozen fresh plasma - Full heparinization with a target aPTT of 45-60

Optimized intensive care therapy has improved survival rates,^3^ increasing the need for plastic surgery for the treatment of skin soft-tissue defects. Surgical debridements are performed with the intent of preserving the greatest possible length and best possible functionality of the affected extremities. An early and prophylactic fasciotomy aimed at preventing hypoperfusion-triggered compartment syndromes and the loss of parts of the limb has not proved advantageous and early amputation of a limb does not affect survival rates.^8^

Therefore, a gradual and appropriate necrectomy preserving functional structures should be performed, even if associated with prolonged intensive care and hospitalization.^9^ Considering the quality of life of patients who develop DIC, it is dependent on the extent of the disease and the prognosis based on other secondary diseases and the development of further organ dysfunctions.^2^^,^^10^ Surveys have shown that the quality of life of patients with DIC can be improved by early treatment of the underlying disease as well as early treatment of the DIC itself. These results clearly show that the quality of life of patients with DIC can be improved by early treatment of the underlying disease and the DIC itself.^2^^,^^10^

However, after survival and completion of intensive care and surgical therapy, the supply with prostheses is necessary. Due to constant developments and optimizations in terms of signal processing and functionality, modern stump restoration after a necessary amputation now offers versatile options for supplying myoelectric prostheses. The prosthetic restoration of transradial amputee patients is relatively simple compared to that of patients with higher amputation levels. Technical progress in the field of prosthetics and the diversity of acceptance rates of myoelectric prostheses has led to increasing demands on the patient, the stump, and ultimately functionality.^11^

Complex surgical amputation techniques, including the creation of the Agonist-Antagonist Myoneural Interface (AMI)^12^ and the development of Regenerative Peripheral Nerve Interface (RPNI)^13^ have been established, leading to the optimization of signal transmission and decoding. However, control options have advanced significantly in recent years allowing for the human-machine interface pattern recognition and regression method applied to the surface EMG to control the multi-degree of freedom (DOF) motor tasks intended by the user.^14^

While these methods are effective under certain conditions, they still have some disadvantages. Implementing multiple simultaneously occurring degrees of movement is a current not readily achieved objective.^15^ By pattern recognition systems of myoelectric signals, the control of a prosthetic hand with multiple grip forms can be improved. This requires clear EMG signals. In the context of sharp amputations caused by traumatic events, the skin soft tissue mantle remains uninvolved and can be used for signal derivation.^15^

Despite the prosthetic development, body image and self-perception as well as quality of life are changed after amputation. The association with self-disgust and prosthesis use after amputation was analyzed in a sample with focus on lower limb amputations. It was recognized that self-rejection was correlated significantly with the frequency of prosthetic use and also the frequency of prosthetic use was significantly positively associated with quality of life. By correcting the body sheath, prosthetic supply has positive psychological benefits that go beyond functional added-value, presumably beneficial for the long-term psychological adaptation of those with lower extremity amputations with a clear reduction in the rates of depression.^16^^-^^18^

However, for the application of the prosthesis both functionality and comfort as well as the integration in the body image play a significant role in prosthetic application. In the context of DIC occurring with damage and necrosis of the skin soft-tissue mantle, we hypothetized an impairment in signal processing and with it the control possibility of the prosthesis, which is not proven in previous studies so far. The clinical parameters of amputation surgery were examined retrospectively and amputations analyzed in terms of extent, frequency and distribution pattern, including required re-amputations. A clinical follow-up survey was used to evaluate functionality and quality of life after bionic prosthetic restoration.

## MATERIALS AND METHODS

A monocentric, retrospective analysis of the surgical course of patients with DIC from 2015 to 1018 was performed. We retrospectively studied parameters from the onset of symptoms to surgical intensive care unit admission as well as the duration of intensive care therapy. We also analyzed the interval from symptom onset to the initial upper and lower extremity necrectomy. Furthermore, the necessity of re-amputation as well as further operations to restore the skin soft-tissue mantle and the final amputation level of the different locations of the upper and lower limbs were assessed.

Clinical follow-up of bionic prosthesis care was performed using the metrics of (a) the Disabilities of the Arm, Shoulder and Hand (DASH) score, (b) the Box and Blocks Test (BBT), (c) Graded Chronic Pain Status (GCPS), (d) the Short Form 36 (SF-36), (e) the Trinity Amputation and Prosthesis Experience Scales-Revised (TAPES-R), and (f) the Wolf Motor Function Test (WMFT). The bimanual tasks and limitations in daily life were rated with DASH score, while a score of 100 indicated the worst hand function and 0.00 as the best. The questionnaire evaluated complaints and problems with everyday activities caused by the arm, shoulder, or hand.^19^

BBT, a functional test of the upper extremities, was performed to measure the gross hand skills of the patient, in particular those with a prosthesis of the upper extremities. The test was consisted of a box with a partition in the middle. During a run, the patient had 60 seconds to move as many blocks as possible from one side to the other using only the hand being tested. The number of moved blocks was a measure of gross manual dexterity, with a higher number indicating better gross proficiency. Reference values ​​were derived from healthy volunteers or from experiments performed with a prosthesis. The box and block test assessed the progress of manual labor during rehabilitation.^20^^,^^21^

The GCPS was a proven instrument for classifying the severity of pain chronicity. The GCPS was based on a questionnaire that classifies pain-related impairment. There were one to four degrees of chronicity based on impairment points. Grades I and II indicated functional persistent pain (in the sense of minor impairment or impairment of function), while grades III and IV indicated dysfunctional chronic pain (severe impairment or impairment of function).^22^

The SF-36 generated an 8-level profile of functional health and well-being values ​​for physical and mental health to estimate the burden of disease. The SF-36 provided a general assessment by calculating values ​​for both physical and mental health. Scores ranged from 0.00 to 100 points, with 0.00 indicating the greatest possible restriction of health and 100 indicating the absence of health restrictions.^23^ TAPES-R was used as a multidimensional tool for studying the psychosocial processes involved in amputation and prosthetic adaptation. It analyzed four areas of (a) psychosocial adaptation (general adaptation, social adaptation), (b) activity restriction, (c) satisfaction with the prosthesis (functional and aesthetic), and (d) evaluation of phantom pain, body aches, and other diseases not related to the amputation.^24^

The WMFT was used to assess arm-hand motor function, allowing therapists to determine if a patient could handle various tasks and a patient’s quality of movement. This allowed therapy to be individually planned and evaluated. The test measured movement quality and functionality (each on a 0 to 5 scale) and required task time. The test could document the course of therapy and grouping of patients.^25^ All patients agreed to the analysis and publication of the data and images. The approval of this retrospective examination and the follow-up examination of the bionic prosthesis function was granted by the local University Ethics Committee (Ethics number 7603). The research was conducted in accordance with the 1964 Helsinki Declaration. Descriptive metrics and their deviations (95% confidence interval, alpha level of 0.05) were generated. Additionally, a Pearson correlation was performed comparing DASH to BBT and DASH to GCPS using Microsoft Excel (Version 15.14 for Mac, Microsoft Corporation).

## RESULTS

**Fig. 1:**
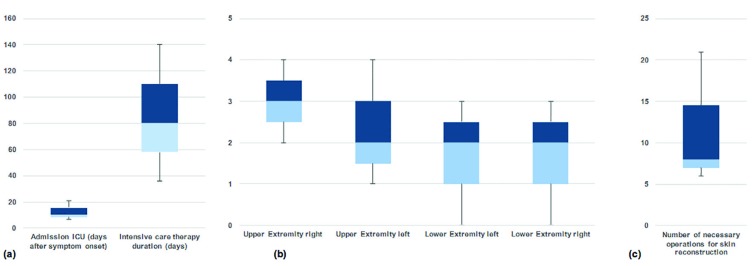
Parameters of surgical and intensive care treatment

We analyzed three patients suffering from DIC after meningococcaemia associated sepsis (2f, 1m). Their average (x̅) and median (x͂) ages, length of time between onset of symptoms and admission to the intensive care unit, and duration of intensive care therapy were shown in Table 2 and Figure 1a. After symptom presentation, the first necrectomy and amputation of the lower extremity was performed after x̅=37 days (x͂=24 days), while the upper limb necrectomy and amputation was performed after x̅=30 days (x͂=24 days). 

**Table 2:** Parameters of surgical therapy ranging from symptom onset to initial necrectomy in the upper and lower limb

**Patient**	**Age**	**Gender**		**Disease**	**Admission ICU (days after symptom onset)**	**Intensive care therapy duration (days)**	**Death**		**Final amputation level**	**Number of amputation and re-exzision**				**Number of necessary operations for skin reconstruction**	**Number of operations initial stay**	**Number of operations after discharge**
										**Upper Extremity**		**Lower Extremity**				
		**M**	**F**				**Yes**	**No**		**Right**	**Left**	**Left**	**Right**			
1	50	1	0	meningococci sepsis	10	140	0	1	Metacarpal right Metacarpal left Transtibial right Transtibial left	4	4	3	2	21	22	3
2	19	0	1	Meningococci sepsis	7	36	0	1	Transradial right Transradial left Transtibial right Transtibial left	3	2	1	1	6	6	2
3	67	0	1	Meningococci sepsis	21	80	0	1	Transradial left Metacarpal right Transtibial right Transtibial left	2	1	2	3	8	10	4
Average or total (n)	45	(1)	(2)		13	85	0%	100%		3	2	2	2	12	13	3
Min					7	36				2	1	1	1	6	6	2
Q1					9	58				3	2	1	1	7	8	3
Median or % of Total (%)	50	**(33%)**	**(67%)**		10	80				3	2	2	2	8	10	3
Q3					16	110				4	3	3	3	15	16	4
Max					21	140				4	4	3	3	21	22	4

All patients required re-amputation, and the average number and localization of these procedures were shown in Table 2 and Figure 1b. A gradual and multi-stage approach was performed to preserve the utmost possible extremity function and length. Thus, multiple operations were required to restore the skin soft-tissue mantle (Table 2 and Figure 1c). All three cases had systemic meningococcal infections leading to sepsis, DIC, and severe skin soft tissue necrosis. Examples of the surgical progression and prostheses of patient 1 and 2 were shown in Figure 2 and 3, respectively.

**Fig. 2.1:**
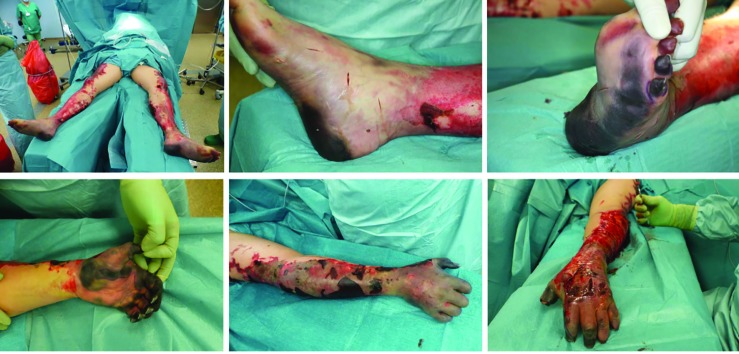
The admission of a 50-year-old patient (Patient #1) was as a secondary acquisition after initial, internal therapy for meningococcal sepsis with the expression of Waterhouse-Friedrichsen syndrome with DIC. Acral necrosis of the hands and feet developed. After demarcation of the acres, a necrectomy of the upper and lower limb was performed

**Fig. 2.2:**
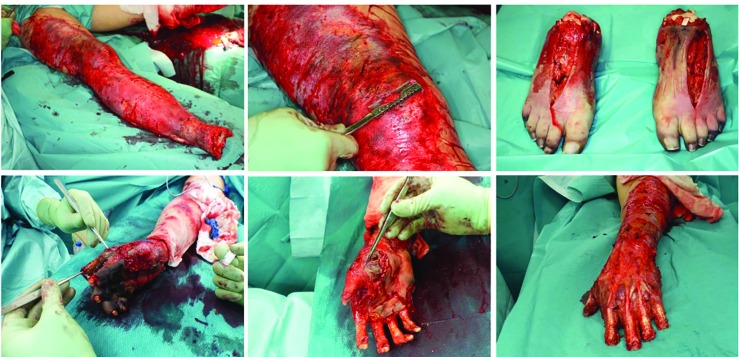
Cross-layer necrosis of the feet necessitated disarticulation of the ankle and feet on both sides. To maximize length, the hands were initially treated by disarticulation in the PIP joint of the Digitus II-V (hand left) and DII/III (right hand). In addition, a necrectomy of the distal lower leg soft tissue was performed

**Fig. 2.3:**
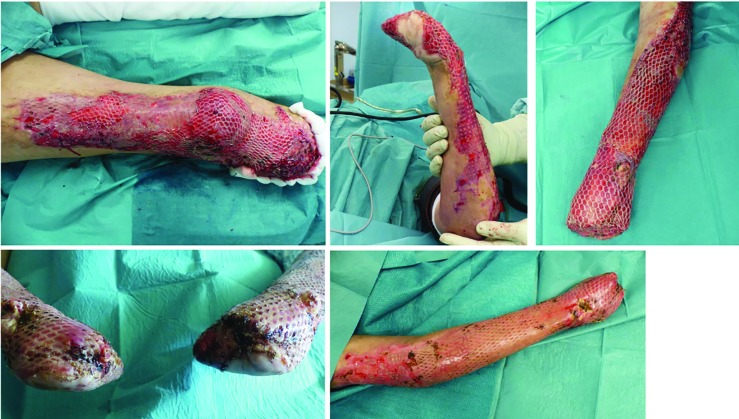
A dermal soft tissue deficit of the stump required a split skin graft according to MEEK, due to the limited donor areas of the back. Also, the skin defects in the thighs and both arms were covered with split thickness skin grafts

**Fig. 2.4:**
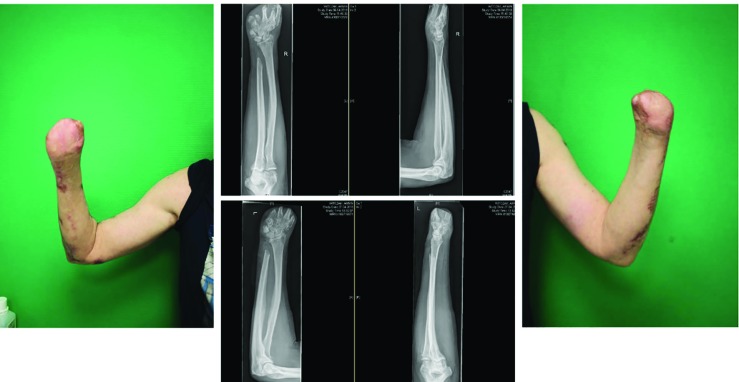
The final amputation level of both upper extremities was located at the level of the metacarpal and transtibial for the lower extremity. After completing intensive care and reconstructive treatment successfully and in good general condition, the patient was discharged to a clinic specialized in amputation medicine for further rehabilitation

**Fig. 2.5:**
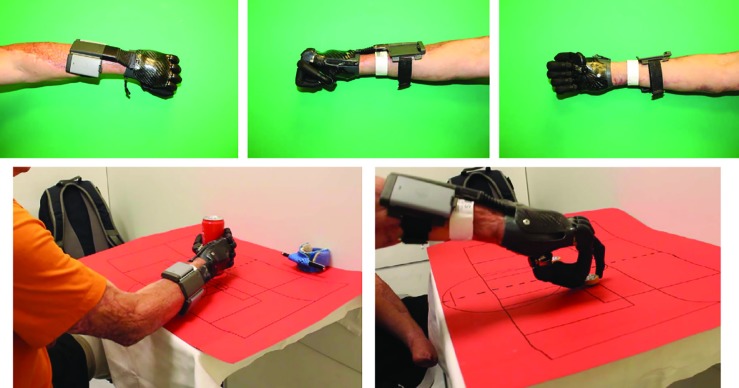
Bionic prostheses supply with Touchbionic, i-digits quantum, which derives signals in the thenar and metacarpal area

**Fig. 3.1:**
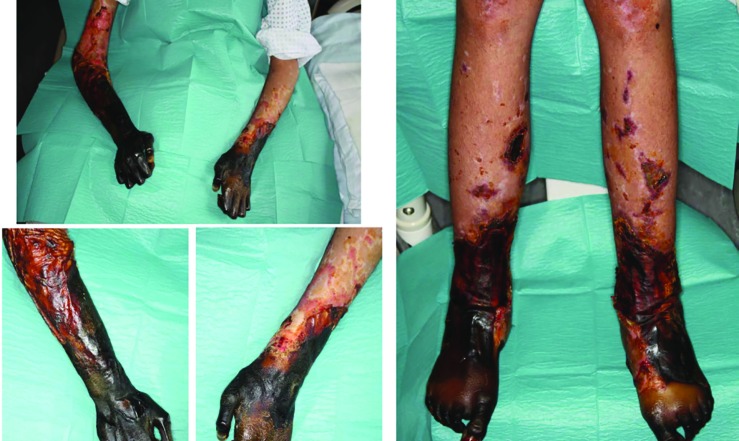
Initially, the patient presented with headache and abdominal pain, which developed on the previous day in an external hospital. With the diagnosis of septic shock, antibiotic therapy was initiated. The clinicians were unable to prevent development of a thombotic microangiopathy of the acres and internal organs. The patient was transferred to the plastic-surgical standard ward for surgical treatment of necrosis of the extremities. In the case of unstoppable necrosis of the hands and feet, the amputation was performed at the middle third of the lower leg on both sides. After serial operations to complete the necrectomies, load-dependent wound healing disorders in both hands and forearms in the skin-clad areas developed. Thus, debridements and split skin transplants had to be performed repeatedly

**Fig. 3.2:**
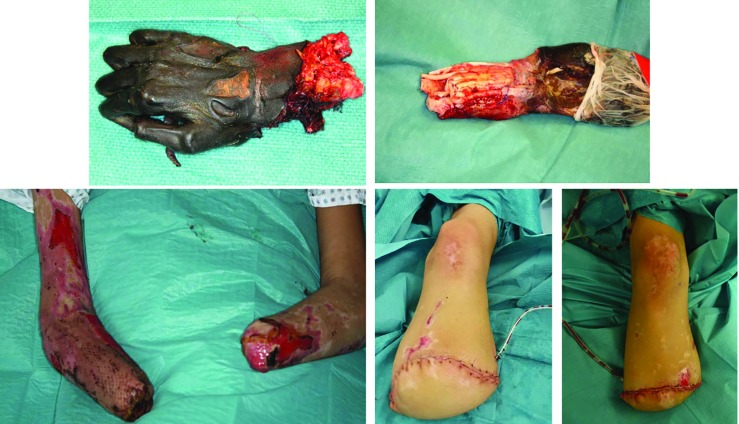
A timely healing tendency with vital split-skin grafts and minimal residual defects was required before discharge. Following discharge, the patient was immediately rehabilitated and prosthetically treated in a special clinic for amputation injuries. As early as 6 weeks after discharge from the acute care clinic, the patient was walking independently and without walking aids. Wound healing disorders of the split-skin grafted areas were minor enough to be treated conservatively and achieve healing

**Fig. 3.3:**
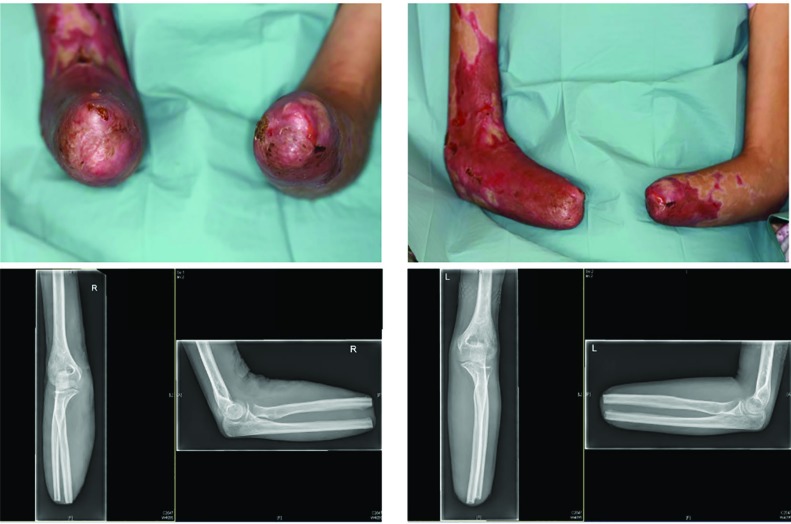
The final amputation levels were trans-radial in both arms and transtibial in both lower legs

**Fig. 3.4:**
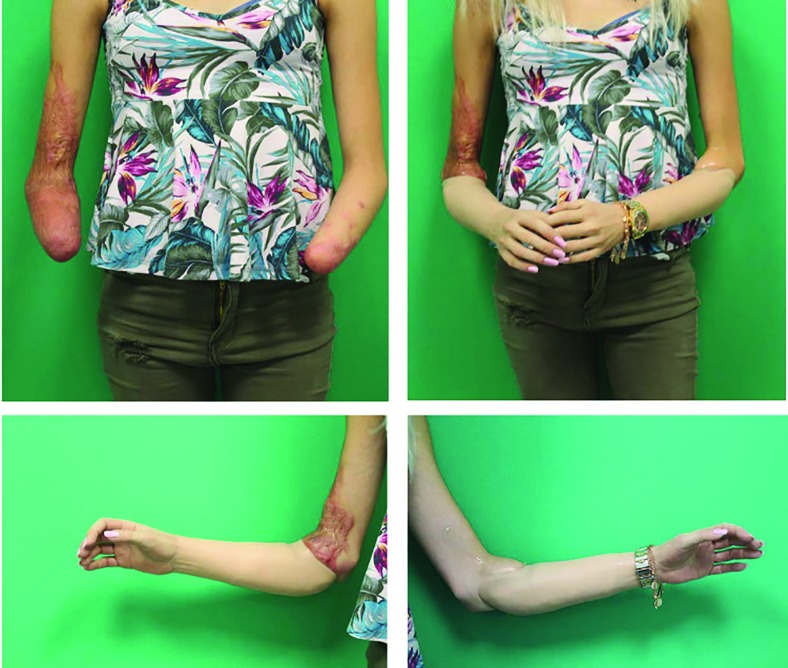
Ottobock, Michelangelo Hand, deriving signals above the proximal forearm

**Fig. 3.5:**
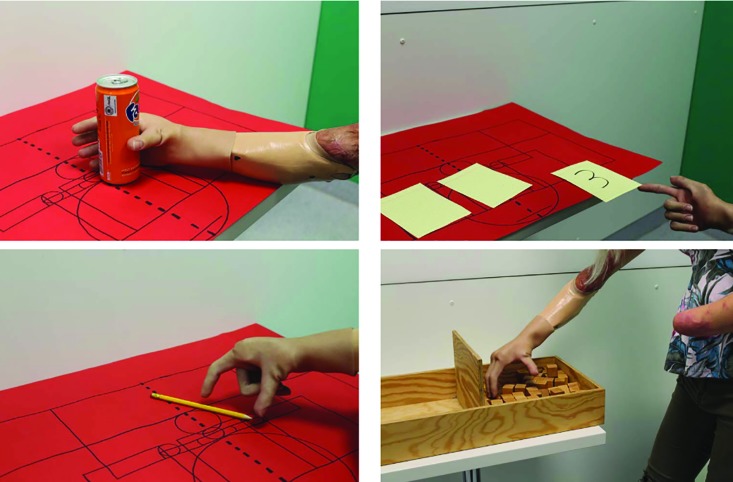
Ottobock, Michelangelo Hand, deriving signals above the proximal forearm

The final amputation levels were located transtibial in the lower extremities and transradial as well as metacarpal in ​​the upper extremities. The following upper extremity prostheses were used: Patient 1: Touchbionic, i-digits quantum, derivation of signals in the thenar and metacarpal area, patient 2: Touchbionic, i-limb quantum, derivation of signals above the proximal forearm, and patient 3: Ottobock, Michelangelo Hand, deriving signals above the proximal forearm.

Regarding the outcome measurements, the average DASH score was 61 points (x͂=61±SD: 5). For BBT, a higher number of blocks moved indicating a better gross proficiency. On average, 14 blocks were placed in one minute (SD**±**12, Min: 0.00, Max: 25). The defined BBT could be considered as an average outcome despite different prosthetic supplies. There was a significant negative Pearson correlation between DASH and BBT (r=-0.99, *p*<0.05) (Figure 4). The measurement of the GCPS indicated a slight impairment, with a pain intensity x̅=17.7 (<50 points, grade I). There was a positive, but not significant, correlation between DASH and GCPS (r=0.98, *p*=0.067) (Figure 4). 

**Fig. 4.1:**
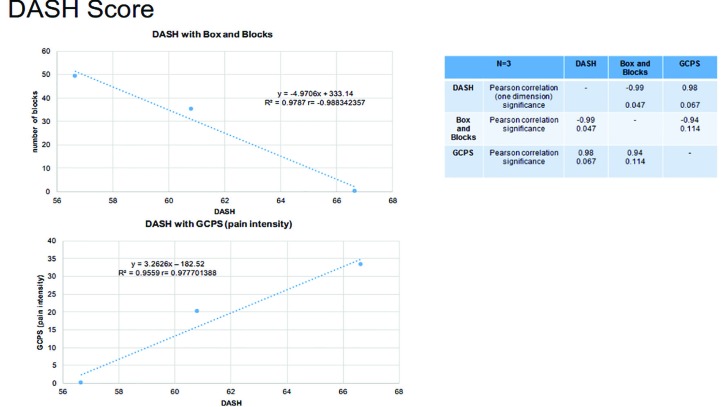
The DASH score had a negative correlation with the BBT and a positive correlation with GCPS**.** The DASH score averaged 61 points (x͂=61±SD: 5)

**Fig. 4.2:**
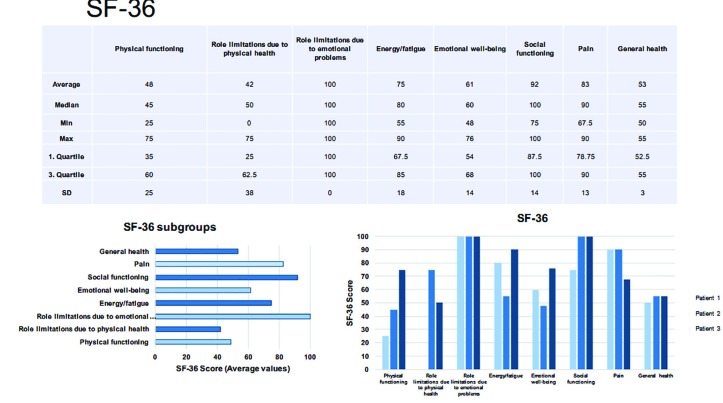
The SF-36 scored an average of 69 points, with 0 points representing the greatest possible health restriction and 100 points the absence of health restriction

Regarding SF-36, the values ​​of the individual measurements were shown in Figure 4.1. Averaging all categories yielded an overall SF-36 value of 69. The TAPES-R results were shown in Table 3. On average, a prothesis was supplied after 8 months post-completion of surgical therapy, which was the time point for the follow up survey (Table 3). The average TAPES-R score for psychosocial adjustment was 2.62, which indicated a poor to fair outcome. The aesthetic satisfaction score averaged 2.2 (on a scale of 1-3), indicating a good aesthetic outcome. The functional satisfaction score (x̅=1.47) indicated an average outcome. The overall average of satisfaction metrics was 4.67, indicating a fair outcome. The physical skills were rated between fair and poor (x̅=2.67). On average, each patient carried their prosthesis 5.67 h per day. Considering the WMFT, x̅=3.87 (±SD: 0.77). The results of the subtests were presented in Table 4.

**Table 3:** The trinity amputation and prosthesis experience scales-revised (TAPES-R). The TAPES-R score for psychosocial adjustment averaged 2.62 points, which indicates a poor to fair outcome. The aesthetic satisfaction score was, on average, 2.2 (on a scale of 1-3), indicating a good aesthetic outcome. The functional satisfaction score was 1.47 (on a scale of 1-3), which indicates an average outcome. Overall a satisfaction score of of 4.67 was reached which is a fair outcome. On average, patients carried their prosthesis daily for 5.67 h

**Study-ID**	**Gender** **Male=1 Female=2**		**How long ago did you have your amputation? (in months)**			**How long have you had a prosthesis? (in months)**			**How long have you had the prosthesis that you wear at the moment**				**What type of prosthesis do you have?**	
Patient 1	1		16			9			9				Metacarpal right/left	
Patient 2	2		19			6			3				Transradial right/left	
Patient 3	2		20			10			10				Transradial left Metacarpal right	
Average			18.3			8.3			7.3					
Min			16			6			3					
Max			20			10			10					
1. Quartil			17.5			7.5			6					
3. Quartil			19.5			9.5			9.5					
**Study-ID**	**Psychosocial adjustment** **(high scores indicative of adjustment)**				**Activity limitation** **(high scores indicative for activity restriction)**		**Satisfaction with prosthesis** **(high scores indicative of satisfaction)**					**On average, how many hours a day do you wear your prosthesis? (hours)**	**In general, would you say your health is?**	**In general, would you say your physical capabilities are?**
	**General Adjustement** **(1-4)**	**Social Adjustement** **(1-4)**		**Adjustement to Limitation** **(1-4)**	**(0-2)**		**Aesthetic Satisfaction** **(1-3)**	**Functional Satisfaction** **(1-3)**		**Satisfaction ** **(0–10)**				
Patient 1	3.2	2.8		1.6	1		1.3	1.2		6	16		4	2
Patient 2	2.4	2.8		2.2	1.1		2.3	1.4		3	0.5		4	3
Patient 3	3	3.6		2	0.3		3	1.8		5	0.5		4	3
Average	2.87	3.07		1.93	0.8		2.2	1.47		4.67	5.67		4	2.67
Median	3	2.8		2	1		2.3	1.4		5	0.5		4	3

**Table 4:** Wolf-Motor-Function Test (WMFT). The WMFT x̅=3.87 (±SD: 0.77), which indicates that arm-hand activities were good

	**Forearm to table**		**Forearm to box**		**Extend elbow**		**Extend elbow with weight**		**Hand to table**			**Hand to box**		**Reach/Retrieve**		**Lift can**	
	**Time in sec.**	**Ability**	**Time in sec.**	**Ability**	**Time in sec.**	**Ability**	**Time in sec.**	**Ability**	**Time in sec.**		**Ability**	**Time in sec.**	**Ability**	**Time in sec.**	**Ability**	**Time in sec.**	**Ability**
Normative values	0.6	5	0.7	5	0.4	5	0.4	5	0.5		5	0.5	5	0.6	5	0.9	5
1	0.8	5	0.6	5	0.7	5	0.6	5	1.5		4	1	4	1.3	3	9.7	2
2	1.2	5	1.4	5	1.5	5	0.8	5	4.3		5	3.2	5	1.5	5	7.6	5
3	1.1	5	3.7	5	7.6	4	3.3	5	1.2		5	2.5	4	3	4	102	2
Average	1.03	5.00	1.90	5.00	3.27	4.67	1.57	5.00	2.33		4.67	2.23	4.33	1.93	4.00	39.77	3.00
Median	1.10	5.00	1.40	5.00	1.50	5.00	0.80	5.00	1.50		5.00	2.50	4.00	1.50	4.00	9.70	2.00
Standard deviation	0.21	0.00	1.61	0.00	3.77	0.58	1.50	0.00	1.71		0.58	1.12	0.58	0.93	1.00	53.91	1.73
	**Lift pencil**		**Lift paper clip**		**Stack checkers**		**Flip cards**		**Turn key in lock**			**Fold towel**		**Lift basket**			
	**Time in sec.**	**Ability**	**Time in sec.**	**Ability**	**Time in sec.**	**Ability**	**Time in sec.**	**Ability**	**Time in sec.**	**Ability**		**Time in sec.**	**Ability**	**Time in sec.**	**Ability**		
Normative values	0.8	5	1	5	2.4	5	2.8	5	1.8	**5**		**2.6**	**5**	**1.6**	**5**		
1	3.9	4	3.3	4	7.2	4	12.8	3	20.9	**2**		**9**	**4**	**8.7**	**3**		
2	42.3	2	40	2	8.3	5	33.6	4	24	**4**		**17.5**	**3**	**7.3**	**5**		
3	-	2	-	2	-	2	69	3	46	**3**		**27**	**3**	**22.4**	**3**		
Average	55.40	2.67	54.43	2.67	45.17	3.67	38.47	3.33	30.30	**3.00**		**17.83**	**3.33**	**12.80**	**3.67**		
Median	42.30	2.00	40.00	2.00	8.30	4.00	33.60	3.00	24.00	3.00		17.50	3.00	8.70	3.00		
Standard deviation	59.15	1.15	59.67	1.15	64.81	1.53	28.41	0.58	13.68	1.00		9.00	0.58	8.34	1.15		

Evaluation: 1 very poor, 2 poor, 3 fair, 4 good, 5 very good

## DISCUSSION

DIC is a severe and complex disease that challenges a surgeon’s ability to decide on the reconstructive options required to produce the best possible post-amputation aesthetic and functional outcome. Interdisciplinary treatments seek to minimize the extent of amputations and strive for the restoration of the skin and soft tissue layers to yield optimal prosthesis adjustment. Regarding the outcome measurements, for BBT, a higher number of blocks moved indicating a better gross proficiency. The results could be compared with reference values ​​of healthy volunteers or reference values ​​of experiments performed with a prosthesis. On average, 14 blocks were placed in one minute. A previous study has shown for experienced myoelectic prosthesis users a BBT score of 13.69±0.84.^26^ For healthy people normative scores showed a BBT score of of 84.9±8.2 blocks.^20^ The defined BBT could be considered as an average outcome despite different prosthetic supplies. 

The complexity and duration of intensive care therapy and restoration of the skin soft tissue mantle combined with complex dressing changes in analgosedation justified the long inpatient treatment in this study, which extended, on average, to almost three months (Table 2, Figure 1a). For maximum length preservation, a “wait-and-see” approach until extensive demarcation may be beneficial.^27^ Nevertheless, there are also clinical approaches that favor early surgical intervention to prevent superinfection and complications.^28^ No clinical investigation could prove one of the approaches to be beneficial.On average, these patients underwent necrectomy and amputation of the lower extremity at 37 days (x͂=24 days) and of the upper extremity at 30 days (x͂=24 days). All patients required re-amputations (Table 2, Figure 1b). With the goal of maximizing the maintenance of autologous tissue, the wait-and-see strategy relies on after-amputation results. 

Using this process in this study, the final determination of the amputation level was made after definitive demarcation or exhaustion of the potential for regeneration. Amputation surgery must be placed in the context of the interdisciplinary and complex therapy of the underlying disease. A variety of metrics were used to evaluate post-amputation functionality and quality of life in patients with prostheses. Their DASH scores had a negative correlation with BBT and a positive correlation with GCPS (Figure 4), so poor functionality (as measured by DASH) corresponded to the movement of fewer blocks in a defined time and a greater experience of pain. 

However, generally, the average GCPS showed only slight impairment. For better evaluation of movement quality the modified BBT should be used in further studies as compared to the classic BBT, not only the rough dexterity but also the quality of the movement patterns can be detected.^29^ The SF-36 indicated some health restrictions (Figure 4.1), but interestingly, no limitations due to “emotional problems”. “Social functioning“ was also high (Figure 4.1), while patients reported only sufficient physical function. 

In contrast, the TAPES-R score for psychosocial adjustment indicated a poor to fair outcome (Table 3). The test methods used may reflect different aspects and therefore, give conflicting results. The SF-36 is not validated as a result variable for prostheses and may therefore not be representative.^30^ Patients were, in general, satisfied with the appearance of their prostheses, and reported average function based on the TAPES-R aesthetic and functional satisfaction scores, respectively. Combining these metrics indicates fair overall patient satisfaction, but physical skills were rated between fair and poor. 

The WMFT indicated patients were in aggregate good at performing arm-hand activities (Table 4). DIC is a serious disease that complicates adaptation to bionic prostheses because of skin soft-tissue damage and consecutive large-scale scarring from surgical recovery and secondary wound healing. Patients complained primarily of uncontrollable prostheses, indicating signal transmission disorders. Due to these signal disturbances, the prostheses often failed based on the large-scale scarring areas. In addition, patient feedback indicated the desire for improvements in mode switching and reductions in the weight of the prosthesis and volume of the servo. One patient indicated dissatisfaction with the weight of the prosthesis, which made it difficult to attach independently. In this case, attachment required constant assistance. 

For optimal use, a light prosthetic weight is essential so that a simple coupling to the stump is possible. Too heavy prosthesis is one of the main reasons for non-use of myoelectric prostheses. Based on the human hand of the dural wrist weighing about 400 grams, moyoelectric devices of similar weight are considered too heavy by users.^31^ The reason lies in an inefficient force transmission. Solutions for optimizing the weight are, for example, osseointegrated prostheses, which offer optimum force transmission.^32^^,^^33^

In addition, many different materials are used in research approaches, especially in the field of 3D printing becoming a viable alternative for customized myoelectric prosthesis. For example, acrylonitrile butadiene styrene (ABS), polylactic acid (PLA) and thermoplastic polyurethane are used for a lightweight and inexpensive production.^34^ For another patient, multiple adjustments were necessary. Over the course of 9 months, adjustments were necessary repeatedly. Shaft flexibility should also be optimized to limit stress conditions and pain. The above deficits limited the daily use of the prostheses to an average of 5.67 h. 

Use of implantable electrodes to record EMG signals is a sensible option for improving transmission deficits. Pasquina *et al.* showed that IMES (®) is a reliable technology for detecting and wirelessly transmitting EMG signals from remaining muscles to intuitively control a three-degree of freedom prosthetic arm.^35^ Due to the gradual and multi-stage approach aimed at achieving the greatest possible function and length preservation of the extremities, an average of 12 operations to restore the skin soft-tissue layers were necessary (Table 2 and Figure 1c). 

These included the entire armentarium of plastic reconstructive surgery from debridement to local muscle flaps. Other additive therapies, including hyperbaric oxygenation, could be considered. This was not performed for the examined patients due to lack of availability and limited evidence of efficacy. To date evidence based controlled randomized clinical studies that have demonstrated a positive impact of hyperbaric oxygenation on survival rates or progression of DIC do not exist. However, published case reports have shown positive effects of hyperbaric oxygenation (HO) as an adjuvant therapy starting at the first to fifth day after symptom onset for 45 minutes twice daily at 1.5 atmosphere absolute (ATA) and after a two-day break once a day at 1.8 ATA for 60 minutes.^36^

Under this protocol, a complete healing of necrotic skin and subcutaneous tissue could be achieved based on increased oxygen concentration during HO therapy promoted spontaneous wound healing.^36^ For this approach, a close collaboration between surgeons and hyperbaric medicine facilities is essential for proper treatment.^37^ This study’s multi-stage approach required long hospitalization, meaning a delay in inpatient rehabilitation for the patients. Long stays are associated with respiratory tract and other organ system complications.^38^

Being bed-ridden for a long period and having immobilized large joints, often leads to critical illness polyneuropathy (CIP) and myopathy (MIP) with muscle atrophy based on axonal degeneration and myosin loss caused by microcirculatory abnormalities, metabolic derangements, reversible channelopathy, and bioenergetic dysfunctions.^39^ After reconstruction of the soft tissue, wound healing disorders and unstable and/or hypertrophic scars, as well as scar contractures complicate the postoperative course of treatment and rehabilitation and require further corrective interventions.^38^

Impaired skin integrity complicates the subsequent prosthetic fitting and undisturbed wound healing of the stumps is required before prosthetic fitting can occur. Prosthesis adaptation requires stable skin soft tissue in the stump area and regular checks of the prosthetic fit, including the stump.^38^ Nevertheless, individually pressure-adapted prosthetic stems make sense. After inpatient treatment, intensive rehabilitation is vital. In this context, prosthetic adaptation is carried out with functional training using the prosthesis. 

In conclusion, DIC is a complex diseases with devastating tissue damage. Patients often emerge from treatment with multiple amputations and require extensive rehabilitation in specialized rehabilitation centers. Functional disadvantages associated with prostheses include a loss of body sensation, prostheses weight, signal transmission disorders, and repeated functional failures. For patients with extensive scarring in the stump area after DIC, implantable signal electrodes (IMES electrodes) may improve signal transmission of bionic prostheses.
